# Diabetes education improves eating habits and quality of life in type 1 diabetes in carbohydrate counting

**DOI:** 10.1186/1758-5996-7-S1-A166

**Published:** 2015-11-11

**Authors:** Débora Bohnen Guimarães, Juliana Bohnen Guimarães, Marcella Lobato Dias Consoli, Janice Sepúlveda Reis

**Affiliations:** 1 Instituto de Ensino e Pesquisa da Santa Casa de Belo Horizonte, Belo Horizonte, Brazil

## Background

Counting carbohydrates, nutritional therapy of choice to type 1 diabetics (DM1), allows flexibility in food choices, avoiding diets based on restrictions, and patients can use any food within a healthy eating plan. With an emphasis on the amount of carbohydrates, a change in the quality of the menu, which can lead to inclusion of foods with high glycemic index and high in fat, is often observed, frequently causing weight gain and worsening of lipid profile. Educating and motivating type 1 diabetics in carbohydrate counting to follow continuously the healthy eating plan is a big challenge. In diabetes education group, reinforcing the theory of good nutrition along with carbohydrate counting, with the aim of facilitating the daily planning meals or food exchanges in social gatherings, can be a strategy to improve adherence to nutritional treatment.

## Objective

The purpose of this study was to evaluate the effectiveness of diabetes education in adherence to healthy eating plan and quality of life in patients with type 1 diabetes in carbohydrate counting.

## Material and methods

Sixteen patients with DM1 in carbohydrate counting underwent a nutrition education program on diabetes during four weeks and then reassessed at 1 and 3 months. Anthropometric and biochemical (A1C e lipid profile) test Results, total daily dose of insulin, and adherence to plan healthy food were analyzed; questionnaires about healthy eating, calculation of 3-day food record, and quality-of-life (Problems Areas in Diabetes–Brazil [B-PAID]) were also assessed.

## Results

Before and 1 and 3 months of the project, an increased adherence to healthy eating plan was demonstrated, with a significant decrease in caloric intake (p=0.008), carbohydrates (p=0.001), and lipids (p=0.04); an improvement in lifestyle habits like eating fruits, foods with sugar or sweets and lower supply frequency in fast foods; and improved overall reading nutrition label and proper treatment of hypoglycemia (p < 0.05). Improvement on the scale of quality-of-life B-PAID was observed during the 3 periods (p=0.001). There was no difference in anthropometric and biochemical tests between periods.

## Conclusion

This study demonstrated that a program of nutritional education in diabetes group for T1D in carbohydrate counting was effective in increasing adherence to healthy eating plan and improving the quality-of-life indices.

**Figure 1:**
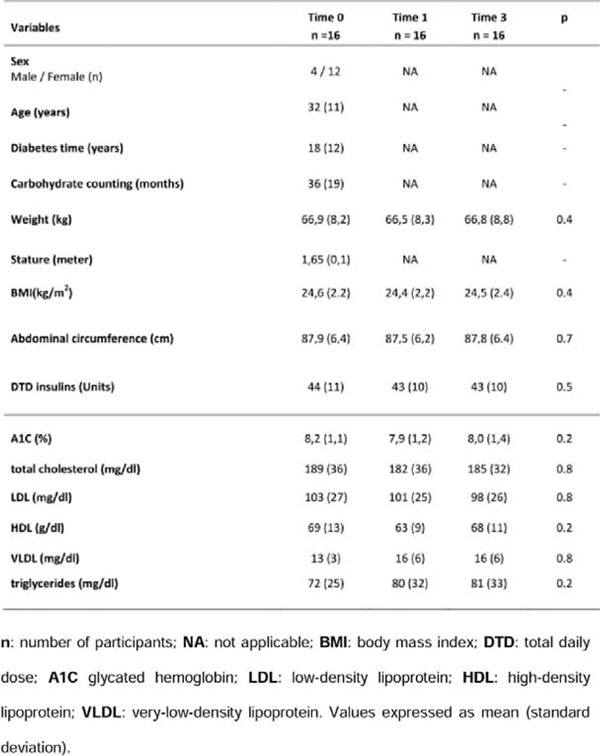
Clinical, anthropometric and biochemical data of patients.

**Figure 2:**
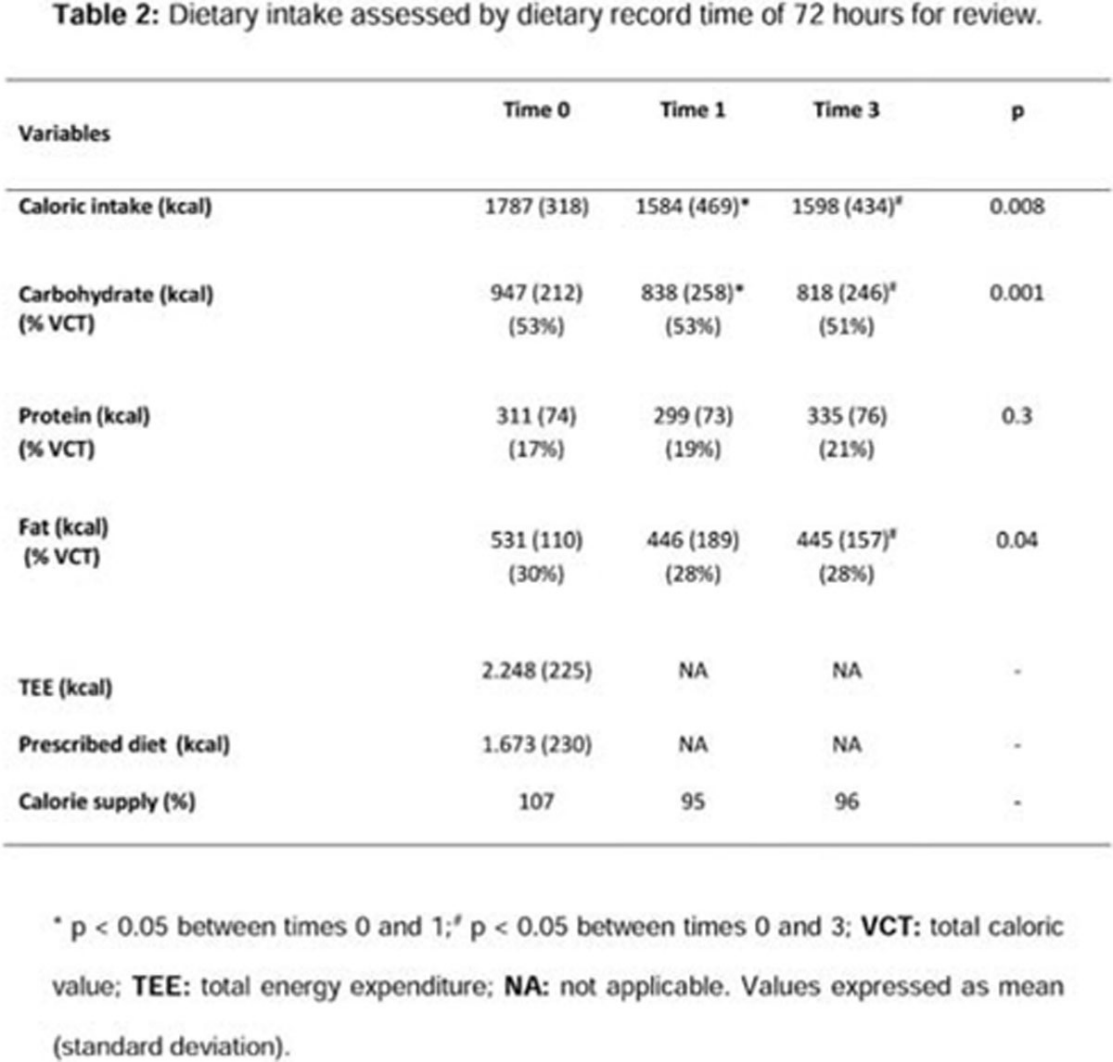
Dietary intake assessed by dietary record time of 72 hours for review.

**Figure 3:**
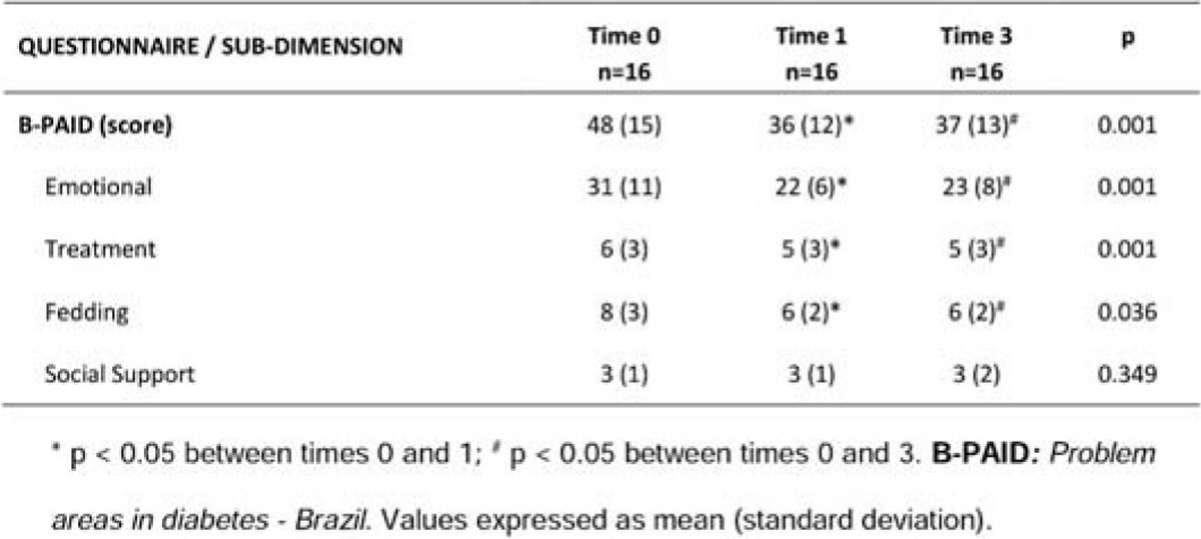
Score on the questionnaire B-PAID and its subdimensions for the evaluation of time.

